# Energy efficiency trade-offs drive nucleotide usage in transcribed regions

**DOI:** 10.1038/ncomms11334

**Published:** 2016-04-21

**Authors:** Wei-Hua Chen, Guanting Lu, Peer Bork, Songnian Hu, Martin J. Lercher

**Affiliations:** 1 CAS Key Laboratory of Genome Sciences and Information, Beijing Institute of Genomics, Chinese Academy of Sciences, Beijing 100101, China; 2 Structural and Computational Unit, European Molecular Biology Laboratory, Heidelberg 69117, Germany; 3 Bioinformatics department, Max Delbrück Centre for Molecular Medicine, Berlin 13125, Germany; 4 Institute for Computer Science and Cluster of Excellence on Plant Sciences, Heinrich Heine University, Düsseldorf 40225, Germany

## Abstract

Efficient nutrient usage is a trait under universal selection. A substantial part of cellular resources is spent on making nucleotides. We thus expect preferential use of cheaper nucleotides especially in transcribed sequences, which are often amplified thousand-fold compared with genomic sequences. To test this hypothesis, we derive a mutation-selection-drift equilibrium model for nucleotide skews (strand-specific usage of ‘A' versus ‘T' and ‘G' versus ‘C'), which explains nucleotide skews across 1,550 prokaryotic genomes as a consequence of selection on efficient resource usage. Transcription-related selection generally favours the cheaper nucleotides ‘U' and ‘C' at synonymous sites. However, the information encoded in mRNA is further amplified through translation. Due to unexpected trade-offs in the codon table, cheaper nucleotides encode on average energetically more expensive amino acids. These trade-offs apply to both strand-specific nucleotide usage and GC content, causing a universal bias towards the more expensive nucleotides ‘A' and ‘G' at non-synonymous coding sites.

## Abstract (web summary)

Substantial cellular resources are devoted to nucleotide biosynthesis. Here the authors propose that transcribed regions prefer ‘cheaper' nucleotides, which appears true for synonymous sites, although more expensive nucleotides coding for cheaper amino acids are selected for at non-synonymous sites.

## Graphical abstract

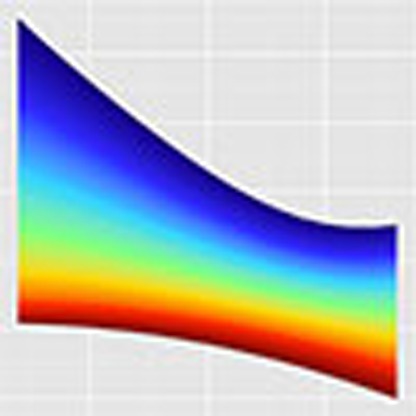

Biochemical energy in the form of adenosine triphosphate (ATP) is a central cellular currency. While some environments provide energy in excess that needs to be dissipated in energy-spilling reactions^1^, most organisms are likely under strong selection for efficient energy usage; many microbes even live under extreme energy limitation^2^. Universal resource-saving measures have been observed to shape various genomic aspects; for example, highly expressed proteins are shorter^3^,^4^ and use cheaper amino acids^5^,^6^,^7^,^8^ than lowly expressed proteins, and microbes preferentially use cheaper amino acids in excreted proteins^9^.

Prokaryotes spend a substantial fraction of their production capacities on making nucleotides—about 13% of *Escherichia coli* glucose consumption is channelled into making nucleotides (see the ‘Methods' section). The synthesis of the five nucleotides adenine, cytosine, guanine, thymine and uracil (A, C, G, T and U, respectively) requires different amounts of energy and nitrogen: *de novo* production costs are A>U/T, G>C and G+C>A+T/U (see the ‘Methods' section)^10^. We expect selection to favour the use of the cheaper over the more expensive nucleotides.

Resource availability varies strongly across environments. That genome composition is under environmental influence is illustrated by a systematic variation of GC content^11^ and synonymous codon usage^12^ across metagenomics data sets from different sources, and by the observation that obligatory pathogens or symbionts tend to be GC poor^13^. While these previous observations were hypothesized to result from mutational biases^11^ and host/symbiont competition^13^, respectively, they may well be a consequence of variable strength of selection on efficient resource usage.

RNA transcripts are often amplified many thousand folds compared with genomic DNA; *E. coli*, for example, spends six times as much cellular resources on making RNA than on making DNA (see the ‘Methods' section). We thus hypothesized that selection for cheaper nucleotides should be most easily detected by comparing transcribed with non-transcribed DNA. Moreover, the fact that usually only one strand is transcribed provides a natural control for GC content when contrasting the transcribed with the complementary untranscribed strand; this approach is equivalent to considering the nucleotide ‘skews' of A versus T and G versus C in transcribed sequences. We predict that transcribed sequences should preferentially use the cheaper nucleotides T/U and C over their respective complementary nucleotides on the strand that corresponds to the RNA produced (that is, the ‘sense' stand).

Nucleotide skews have also been observed in non-transcribed DNA. No single satisfactory explanation of skews and their diversity exists^14^; prokaryotic skews were predominantly attributed to mutational biases associated with the mechanics of leading and lagging strand replication^15^,^16^. To quantify any selection on nucleotide usage associated with transcription, we thus need to disentangle replication-associated mutational skew contributions from the contributions caused by transcription-associated selection.

Here, we derive a mutation-selection-drift model for nucleotide skews and apply it to different types of sites across 1,550 prokaryotic genomes. At fourfold-degenerate sites on coding strands, selection favours cheaper nucleotides, as predicted. At non-synonymous (NS) sites, cheaper nucleotides tend to encode more expensive amino acids; here, selection on amino acid cost prevails, resulting in a strong preference for more expensive nucleotides. We thus provide a unified model for the evolutionary causes of site-type-specific nucleotide skews in prokaryotes. The observed patterns are consistent with strong and ubiquitous selection due to the energetic cost of nucleotides and amino acids, but cannot be explained by mutational biases.

## Results

### Strand identity and the definition of nucleotide skews

There are two different relevant definitions of strand identity: the mutational effects of replication depend on the strand's position relative to the origin of replication (leading versus lagging), while the effects of transcription-associated selection depend on the local direction of transcription (Supplementary Fig. 1). We define AT skew, *γ*_AT_, as the fraction of A minus the fraction of T among all AT basepairs at the strand and type of site considered; likewise, GC skew, *γ*_GC_, is the fraction of G minus the fraction of C among all GC basepairs. Thus, if *γ* is the site-type-specific skew on one strand, then −*γ* is the skew on the complementary DNA. In the absence of systematic biases, we expect *γ*=0. The hypothesis of synthesis cost-driven nucleotide usage bias predicts negative AT and GC skews on sense strands, as A and G are more expensive than T/U and C, respectively (see the ‘Methods' section, Supplementary Data 1)^10^.

### A mutation-selection-drift model for nucleotide skews

We initially focus on fourfold-synonymous (4s) sites of protein-coding genes, where nucleotides can change without compromising the protein function. We assume that GC and AT basepairs are at equilibrium, and only consider mutations that convert A↔T and G↔C.

Let *u* be the mutation rate from C to G on the leading strand (predominantly caused by replication), and *v* the corresponding mutation rate from G to C, each measured per site and per generation. At 4s sites, we need to combine mutational biases with selection on nucleotide usage. Let *s* be the fitness difference between alleles G and C at a given site. Mutation rates are likely much lower than the inverse effective population size, 1/*N*_e_, for most prokaryotes^17^, and thus most sites are fixed at one nucleotide^18^. A standard population genetics model^18^ (see see the ‘Methods' section for details) then predicts the skew on the leading strand as:


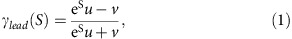


where *S*=2*N*_e_s with effective population size *N*_e_. On the lagging strand, *u* and *v* switch places, and thus


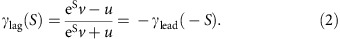


For non-transcribed sites, we assume that *S*=0, and we obtain=*μ* , with the relative skew in mutation rates 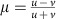. Thus, the skew expected due to mutational biases alone equals the relative difference in mutation rates. The same equations hold for AT skew if we interpret *u* as the mutation rate from T to A on the leading strand, *v* as the reverse mutation rate from A to T, and *s* as the selection coefficient favouring A over T in transcribed sequences. Supplementary Figure 2 shows the dependence of *γ* on *S* for different values of *μ*.

If the scaled selection coefficient is small (*S*<1), selection on individual sites is weak and correspondingly hard to detect. However, when pooling many genomic sites, as done for the calculations of *γ*, we gain the statistical power to detect even relatively small values of *S*.

We assume that the mutational bias *μ* of non-transcribed DNA is constant within any given genome, although it may vary between genomes, for example, due to differences in repair enzymes. An interaction between transcription and replication (caused, for example, by collisions between the replication and transcription machineries) may lead to an amplification of the mutational bias in transcribed sequences, quantified by an amplification factor *τ*; we assume that *τ* is a global, species-independent constant determined by basic cell biology. This assumption of a species-independent amplification factor is supported by a strong linear relationship between estimates of mutational bias for non-transcribed and transcribed sequences (see below). In a first approximation, we also assume that the scaled selection coefficient *S* is a genome-wide constant independent of transcription rate (see below for an analysis that explicitly considers transcription rates).

### Selection at 4s sites across prokaryotes

**Figure 1:**
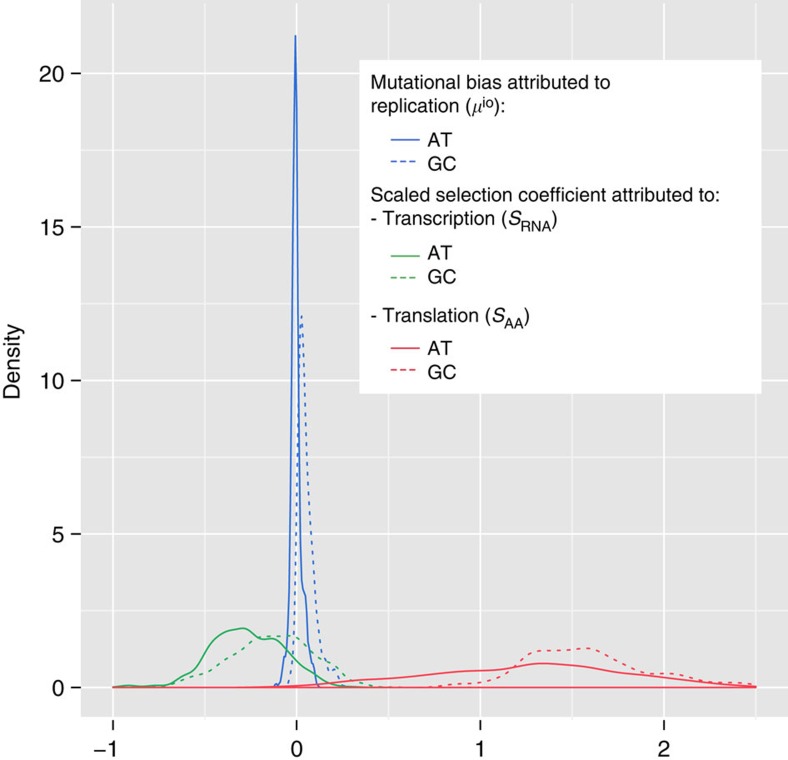
Distribution of mutational bias and scaled selection coefficients that shape nucleotide skews. Solid lines: A versus T; dotted lines: G versus C. Blue: relative difference in mutation rates, *μ*^io^, derived from interoperonic sites; green: selection related to transcription, *S*_RNA_, derived from 4s sites; red: selection related to translation, *S*_AA_, derived from NS sites. Replication-related selection favours the cheaper nucleotides T/U and C in most genomes (*S*_RNA_<0), while translation-related selection almost always favours the more expensive A and G (*S*_AA_>0). The scaled selection coefficients were averaged over the estimates from leading and lagging strand for each genome. The raw/observed skews at different sites are shown in Supplementary Figs 6–8.

To test our hypothesis of global selection on nucleotide usage in transcribed DNA, we applied this mutation-selection equilibrium model to 1,550 fully sequenced prokaryotic genomes. To ensure that our results are not biased by an uneven representation of different phylogenetic clades in public databases, we repeated all analyses on a subset of 344 phylogenetically evenly distributed genomes^19^, with essentially identical results (Supplementary Figs 3–5 and Supplementary Data 2). For each genome, we estimated the mutational bias caused by replication, *μ*^*io*^, as the skew calculated across non-transcribed (interoperonic) regions (Fig. 1; see the ‘Methods' section). In agreement with earlier observations^20^, we find that mutational biases at AT sites on the leading strand are skewed towards T, while those at GC sites are skewed towards G in most prokaryotic genomes: 559 (36.1%) of the 1,550 genomes examined show a higher mutation rate from A to T than vice versa, and 1,456 genomes (94.0%) show a higher mutation rate from C to G than vice versa (Fig. 1).

Contrasting skews across all leading-strand 4s sites (*γ*_lead_) and across all lagging-strand 4s sites (*γ*_lag_) in each genome (Supplementary Data 3 and Supplementary Figs 6–8), we calculated amplified mutational biases for transcribed sequences, *μ*^4s^ (see see the ‘Methods' section):


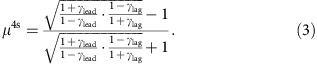


**Figure 2:**
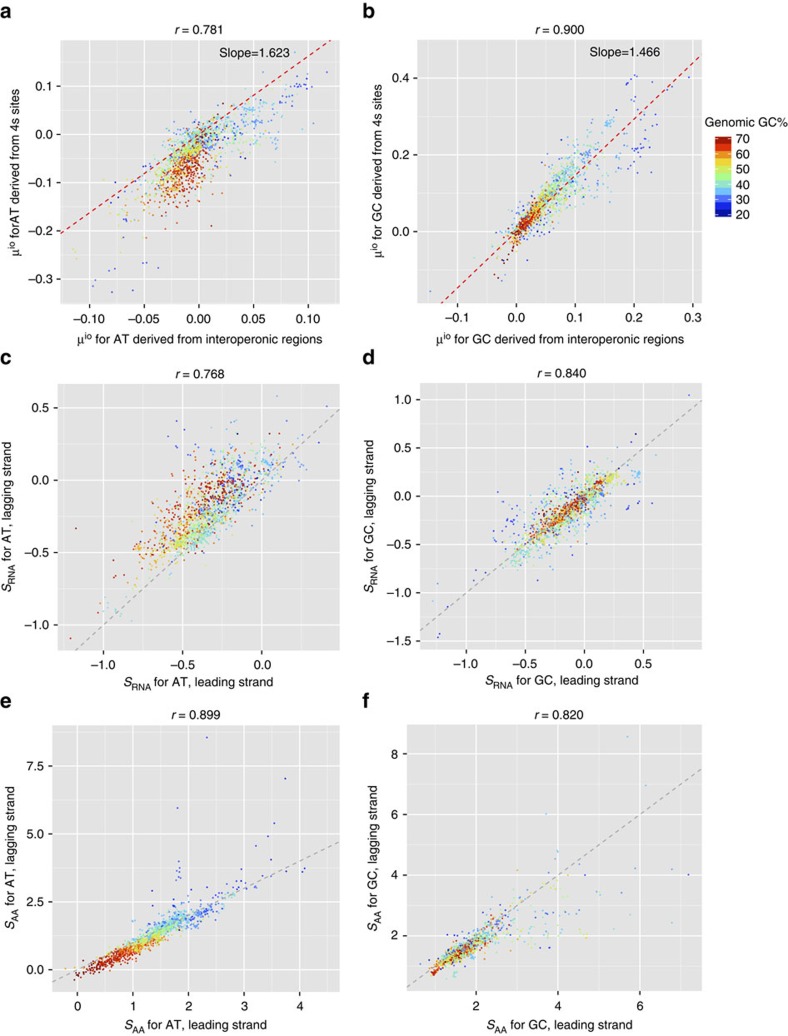
Nucleotide skews are shaped by all three fundamental processes of the central dogma of cell biology. Shown here are mutational biases attributed to replication (**a**,**b**) and scaled selection coefficients attributed to transcription (**c**,**d**) and translation (**e**,**f**), dissected using a mutation-selection-drift equilibrium model. Each point represents one genome, colour-coded based on the genomic GC content. The red diagonal lines in **a** and **b** were obtained using linear regression through the origin; the grey diagonal lines in **c**–**f** indicate the expected identity of the two estimates.

As expected from the assumption of a species-independent amplification of mutational biases in transcribed sequences, we found that the two measures of replication-associated mutational biases derived from non-transcribed and from 4s sites are highly correlated (AT skew: Fig. 2a, Pearson's *r*=0.781, *P*<10^−15^ after controlling for phylogenetic relatedness using independent contrasts^21^; GC skew: Fig. 2b, *R*=0.900, *P*<10^−15^; see Supplementary Figs 6–8 for the raw skews). From these correlations, we determined the global amplification factors *τ*_AT_=1.62 (95% confidence interval=1.54–1.71) and *τ*_GC_=1.47(95% confidence interval (CI)=1.44–1.49).

We then used these global amplification factors to approximate the replication-associated mutational bias at 4s sites, *μ*^4s^, for each genome from the interoperonic estimate. This allowed us to estimate the transcription-associated scaled selection coefficient *S*=2*N*_e_s independently from operons encoded on leading and lagging strands, by solving equations (1) and (2) for *S*:


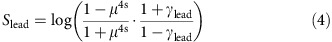



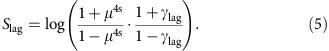


These two estimates are again highly correlated (AT skew: Fig. 2c, *r*=0.768, *P*<10^−15^; GC skew: Fig. 2d, *r*=0.840, *P*<10^−15^). The correlations between the independent estimates for *S* from 4s sites on leading and lagging strands are much stronger than those of the raw observed skews at these sites (*r*=0.502 and 0.392 for AT and GC skews, respectively), thus supporting the validity of our model.

As expected from the hypothesis of preferential usage of cheaper nucleotides in transcribed sequences, we find that *S* is predominantly negative: in the vast majority of the 1,550 studied prokaryotes, the cheaper bases T/U and C are favoured by natural selection relative to their respective complementary bases A and G (Fig. 1; AT skew: *S*<0 for 1,421 genomes (91.7%); GC skew: *S*<0 for 1,108 genomes (71.5%); *P*<10^−15^ from binomial test in each case, also when considering only the subset of phylogenetically evenly distributed species, see the ‘Methods' section).

For GC skew, the observed distribution is consistent with the proposal of a substitution model that incorporates an avoidance of *C* in transcribed sequences^22^. The proposed avoidance of C was interpreted as a response to the different cellular concentrations of the nucleotides: ATP>>GTP>UTP>CTP; this argument cannot be extended to explain the observed dearth of A versus T, and we thus conclude that the need for an additional ‘avoidance' term in the substitution model^22^ was caused by selection for efficient energy usage.

**Figure 3:**
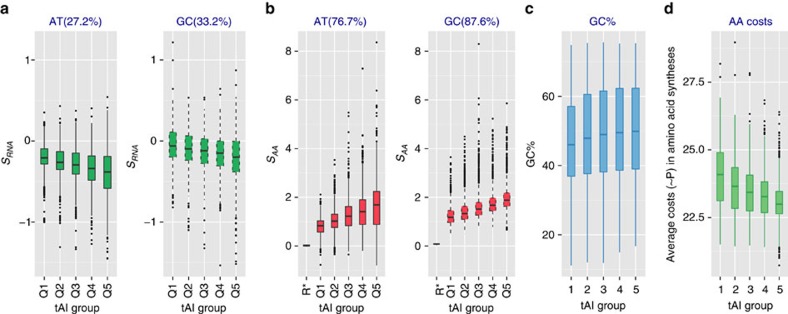
Selection becomes stronger with increasing expression level. tRNA adaptation index (tAI) is used to proxy the expression level; results are plotted as standard boxplot. Scaled selection coefficients on the usage of expensive nucleotides attributed to transcription (**a**) become more negative with increasing expression level, while those attributed to amino acid usage (**b**) increase with increasing expression level. Highly expressed genes also tend to have higher GC content (**c**). Accordingly, average amino acid energy costs (**d**) are much lower in more highly expressed genes. Q1–Q5 contain equal numbers of genes, grouped by expression levels from low to high. Percentages in parentheses give the fraction of genomes in which the skew of genes in the highest expression group (Q5) is higher than that of genes in the lowest expression group (Q1). R* shows the expected distribution for a random data set (see the ‘Methods' section for details). This figure shows data averaged across leading and lagging strands. See Supplementary Data 5 for the averaged amino acid costs of each tAI groups in the 1,550 genomes.

Using tRNA adaptation index (tAI)^23^,^24^ as a proxy for gene expression level, we found that T/U and C become more strongly favoured with increasing expression level (Fig. 3a), as expected due to the stronger amplification of more highly expressed sequences and a resulting stronger selection for cheaper nucleotides.

### Selection trade-off at NS sites

So far, we have only considered the nucleotide skews caused by two of the three fundamental processes covered by the central dogma of cell biology: replication and transcription. How does the third fundamental process, translation, affect nucleotide usage in transcribed sequences? The 20 amino acids differ in the amount of energy needed for their *de novo* production (see the ‘Methods' section and Supplementary Data 4)^5^. An avoidance of energetically expensive amino acids has in fact been put forward as the likely explanation for unusual nucleotide skews observed in one clade of prokaryotes^25^. Is there a systematic relationship between the cost of amino acids and the cost of the nucleotides that encode them; and do the corresponding selection pressures work in unison or oppose each other?

**Figure 4:**
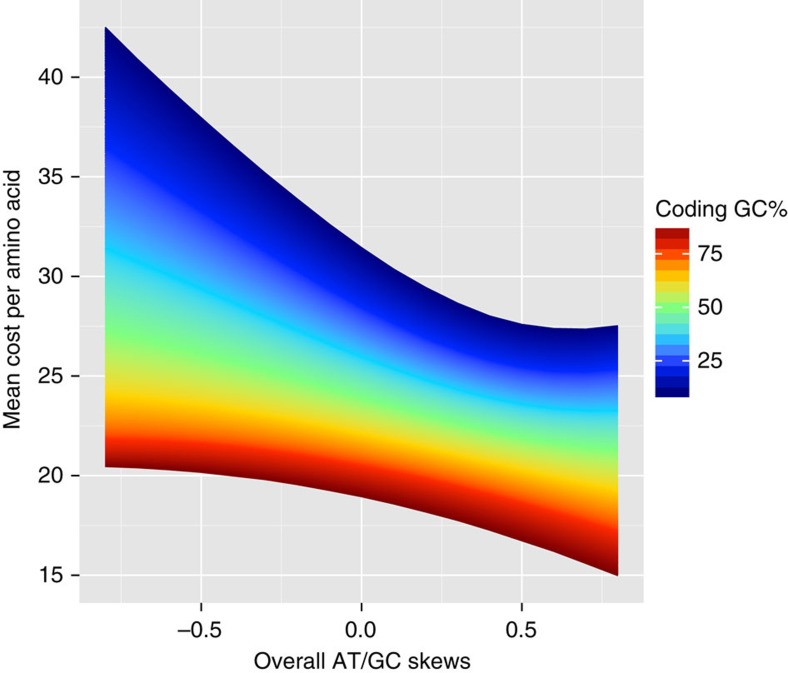
The codon table encodes a trade-off between the energy cost of nucleotides and the energy cost of encoded amino acids. Here the energy cost is measured as the number of high-energy phosphate bonds in ATP molecules used for de novo synthesis; relative costs of RNA nucleotides synthesis are A>U, G>C, and G+C>A+U (see the `Methods' section, Supplementary Data 1)^10^. GC content and skews have independent effects on the costs of amino acids (Supplementary Fig. 9). RNA nucleotide costs increase with increasing skews and GC%; conversely, amino acid costs decrease with increasing GC% (ref. 34) and increasing skew. Thus, increased usage of costly nucleotides results in energetically cheaper amino acids. Each coloured point represents one random coding sequence of given GC content (see colour scale) and given GC and AT skews (position on *x*-axis). See the ‘Methods' section for details.

Calculating the average cost of amino acids encoded by genomes of different GC content and different skews, we find an unexpected trade-off intrinsic to the codon table. RNA production costs increase with increasing skews and GC% (see the ‘Methods' section); conversely, the energy cost of amino acid synthesis decreases both with increasing nucleotide skews and with increasing GC content (Fig. 4). The effects of AT and GC skews are independent (Supplementary Fig. 9). Thus, usage of more costly RNA nucleotides results in energetically cheaper amino acids.

Energy cost varies much more between amino acids than between nucleotides; while the energy needed for the *de novo* production of two RNA nucleotides differs on average by 4.6 ATP units in *E. coli*, the cost difference among amino acids is on average 13.2 ATP units (see the ‘Methods' section). Moreover, each mRNA template will be translated multiple times, amplifying any selection on amino acid usage further: in total, *E. coli* spends 47.3% of its production capacities on amino acids, but only 11.3% on RNA nucleotides (see the ‘Methods' section). Thus, we expect that selection due to translation, which favours expensive nucleotides that encode cheaper amino acids, should dominate the opposing selection for cheap nucleotides at NS sites.

Energy-efficient amino acid usage is of course not the only translation-related selection pressure: many amino acids will be evolutionarily conserved due to selection on protein function. If this selection is unbiased in terms of nucleotide usage, then the simplest way to incorporate selection on protein function into the model is to assume that a certain fraction *f* of amino acid positions evolves neutrally, while the remaining sites are fixed due to selection on protein function^26^ and hence do not contribute to skews. The model can then account for selection on protein function by upscaling the observed skew accordingly when estimating the evolutionary forces acting on NS sites (*γ*→*f*^−1^*γ* in equations (4) and (5)). To obtain a genome-independent estimate of the fraction of free sites, we identify the value of *f* that maximizes the agreement between the mutational bias acting on 4s sites, *μ*^4*s*^=*τμ*^io^, and that estimated at NS sites, *μ*^NS^. We expect the fraction of free sites *f* to be similar between AT positions and GC positions. This is indeed the case: we obtain *f*_AT_=0.311 (95% CI=0.298–0.324) and *f*_GC_=0.348 (95% CI=0.342–0.355; see the ‘Methods' section and Supplementary Figs 6–8).

Selection pressures related to transcription (*S*_RNA_) and translation (*S*_AA_) are independent, and the scaled selection coefficients are thus additive. Hence, we have *S*=*S*_RNA_+*S*_AA_ at the NS sites, and *S*=*S*_RNA_ at the 4s sites. Using the mutational bias derived from interoperonic sites, *τμ*^io^, and the transcription-associated scaled selection coefficient derived from 4s sites, *S*_RNA_, we can use equations (4) and (5) to derive estimates for translation-associated selection on leading and lagging strands for each genome. We find that the two estimates are very similar, further supporting the validity of our model (AT skew: Fig. 2e, *r*=0.899, *P*<10^−15^ after controlling for phylogenetic relatedness; GC skew: Fig. 2f, *r*=0.820, *P*<10^−15)^.

As expected from the trade-off between amino acid and nucleotide synthesis costs, we find that selection related to translation strongly favours the more costly nucleotides A and G: *S*_AA_ derived from AT skews is positive for 1,532 (98.8 %) of the examined genomes, while *S*_AA_ derived from GC skew is positive for 1,435 (92.6%) of genomes (Fig. 1; *P*<10^−15^ in each case from binomial tests, also when considering only the subset of phylogenetically evenly distributed species).

That selection specific to NS sites is indeed related to translation, and is also supported by an analysis of tAI : the translation-associated selection coefficient *S*_AA_ of more highly expressed genes increasingly favours more costly nucleotides (Fig. 3b), likely driven by increased avoidance of expensive amino acids^8^. In addition to increased skew-related selection favouring A over T/U and G over C, highly expressed genes also tend to have higher GC content (Fig. 3c), resulting in a further reduction of the energetic costs of translation (Fig. 3d).

### Mutational biases alone cannot explain the observed skews

Could other, non-selective forces be responsible for the observed differences in skews between transcribed and non-transcribed regions? It has been suggested that nucleotide skews in transcribed regions are due to transcription-associated mutational biases^27^,^28^,^29^,^30^,^31^. During transcription, the nascent mRNA is paired with the anti-sense strand, while the sense strand is unpaired around the site of active transcription^32^. The time spent in the unpaired state during lagging-strand replication is believed to underlie the replication-associated mutational skews; thus, we may expect a similar mutational skew on the sense strand in transcribed regions. Lagging strands in non-transcribed regions are mostly biased towards C and A. While sense strand-specific substitutions are also biased towards C at GC sites, they are predominantly biased towards T at AT sites (Fig. 1). Our observations are thus incompatible with a hypothesis of transcription-associated mutations due to single-strandedness.

Other transcription-associated mutational forces are of course conceivable^27^,^28^,^29^,^30^,^31^. In particular, transcription-coupled repair mechanisms^28^,^30^ might induce nucleotide biases that differ from those caused by replication. However, any nucleotide bias caused by transcription-associated mutation or repair should affect all sites in a given transcribed region equally, and would be unable to distinguish between 4s and NS sites. This, too, is incompatible with our observations, which instead show strong, systematic differences between 4s and NS sites (Fig. 1).

4s and NS sites differ from each other and from non-transcribed sites in their flanking nucleotides, which could potentially bias mutation rates^33^. It is thus conceivable that it is not transcription *per se*, but the specific nucleotide context found around 4s and NS sites that is responsible for the observed skews. However, this ‘genomic context' model is also unlikely to explain the observed skews. First, genomic context-associated mutations would be largely independent of transcription rate, and could hence not account for the observed relationship between skews and expression level (Supplementary Fig. 10).

As a second test whether genomic context could explain our observations, we restricted our analyses to genomic sites with ‘complementary symmetric' neighbours, that is, nucleotides *x* that are flanked by the same two neighbours as their partner on the complementary strand: C*x*G, G*x*C or T*x*A. By construction, genomic context is identical on both strands for these sites, and the genomic context hypothesis predicts no transcription-associated effects. In contrast to this expectation, our analysis restricted to the sites with complementary symmetric neighbours shows transcription-associated selection coefficients that are very similar to those shown in Fig. 1 (Supplementary Fig. 11). We thus conclude that mutational effects are unlikely to explain the observed nucleotide biases in transcribed regions.

### Reduced selection in intracellular symbionts

The metabolic pathways used to synthesize nucleotides and amino acids differ between species, and are often environment-dependent. Such metabolic differences likely contributed to the observed variation in the strength of selection across species. One extreme example is obligate intracellular pathogens or symbionts, species that spend their entire life cycle inside the cells of other organisms—that is, in extremely nutrient-rich environments. We would expect that these intracellular species experience substantially weaker selection on efficient resource usage compared with free-living prokaryotes. This is indeed the case: when comparing a subset of 116 intracellular with 267 free-living species, we found that the strength of selection is significantly reduced for the intracellular species, both for nucleotide usage (AT skew: *P*=1.7 × 10^−4^; GC skew: *P*=2.6 × 10^−5^; *P* values based on a general linear model on phylogenetically independent contrasts^21^) and for amino acid usage (AT skew: *P*=0.000033; GC skew: *P*=0.00116).

## Discussion

From the perspective of efficient resource usage, every mutational change to the genome sequence has fitness consequences. GC-rich codons encode energetically cheaper amino acids^34^, while at the same time G+C basepairs are more expensive than A+T/U pairs (Fig. 4). This trade-off means that high-GC genomes spent proportionally more energy on nucleotide production than low-GC genomes, while the latter spent relatively more on amino acid production. In this sense, GC content is an indicator of relative investment into nucleotides and amino acids^35^. An increased ‘indifference' towards amino acid costs in low-GC genomes is consistent with the observation that AT-rich genomes do not prefer specific amino acids over others to the same extent as GC-rich genomes^35^.

Nucleotide usage in transcribed sequences has mostly been discussed in terms of codon usage bias (CUB)^36^. Consistent with our findings, most preferred codons end with T or C rather than with A or G (Fisher's exact test: odds ratio=2.08, *P*<10^−15^ for T- over A-ending codons; odds ratio=1.68, *P*<10^−15^ for C- over G-ending codons (see the ‘Methods' section). The genomic copy numbers of different tRNAs and their efficiency in recognizing specific synonymous codons constrain the substitution of synonymous nucleotides, and might thus interfere with selection on nucleotide and/or amino acid costs. However, almost all species possess a set of tRNAs capable of recognizing the full set of synonymous codons (often with the help of tRNA anticodon-modifying enzymes^37^). tRNA copy numbers and codon usage are expected to co-evolve^14^,^36^,^38^,^39^ through the accumulation of small changes in both tRNA copy numbers and codon usage in a correlated fashion. Selection on efficient resource usage will put a directional selective pressure on this co-evolution.

Our analysis demonstrates that selection on energetic efficiency, shaped by a trade-off inherent in the codon table, is an ubiquitous feature of prokaryotic genome evolution. The genomic distribution of nucleotides is moulded through an interplay of genomic forces acting on the three levels of the central dogma: mutational biases associated with replication, and energy-driven selection linked to transcription and translation. The same forces likely also play a role in eukaryotic evolution, although their influence may be attenuated by the stronger role of neutral evolution due to the typically smaller effective population sizes of eukaryotes^40^.

## Methods

### Data

Genome sequences and annotations of all completely sequenced bacteria (2,012 genomes as of January 2013) were downloaded from NCBI. In case a genome contained multiple chromosomes, only the largest was chosen.

The genomic coordinates of 2,135 replication origins for 1,712 reference genomes were obtained from the DoriC^41^ database using a PERL web crawler. In case a genome contained multiple replication origins, the first entry in the database was chosen (see Supplementary Data 6 for a list of replication origins used in this study).

Operon predictions were downloaded from DOOR^42^. Because the predictions only cover coding regions, we added other annotated regions including tRNAs and rRNAs from the GFF (General Feature Format) annotations downloaded from NCBI, so that we could extract interoperonic regions, which are presumably non-transcribed. In total, 1,550 genomes were covered by all three data sets (Supplementary Data 3).

The energetic cost (measured as the number of consumed high-energy phosphate bonds contained in ATP and GTP molecules in *de novo* synthesis) for the 20 amino acids in *E. coli* was obtained from ref. 5 (Supplementary Data 4). Synthesis costs in other studied prokaryotes correlate strongly with these values^6^; using alternative costs does not change our qualitative conclusions (Supplementary Data 4 and Supplementary Fig. 13).

The relative costs for the *de novo* synthesis of the four nucleotides have been reported as G>A, C>T, G>C and A>T^10^; see also the calculations for *E. coli* in Supplementary Data 1.

Habitat information of completely sequenced genomes (Supplementary Data 3) was obtained from the NCBI BioProject resource^43^, Integrated Microbial Genome^44^ and Genomes Online Database^45^.

We manually compiled a list of intracellular bacteria (Supplementary Data 7) by searching in public resources, including NCBI PubMed (http://www.ncbi.nlm.nih.gov/pubmed/), Wikipedia (http://en.wikipedia.org/wiki/Intracellular_parasite) and MicrobeWiki (http://microbewiki.kenyon.edu/). The list also contains additional genomes <750 kb in size; this cutoff was chosen because some bacteria of slightly larger size, such as *Mycoplasma pneumoniae* (genome size of 816 kb (ref. 46) are known to be able to grow outside of a host.

### Extracting sequences from interoperonic regions

To obtain regions that are presumably only subject to replication (that is, that are non-transcribed and contain few conserved sites), we extracted interoperonic sequences longer than 100 bp after removing 60 bp adjacent to the 5'-end of genes/operons^25^. If an interoperonic region was located in the second half of the genome (blue dashed line in Supplementary Fig. 1), its sequence was reverse-complemented.

### Strand assignment of protein-coding genes

The division of a genome into leading and lagging strands is shown in Supplementary Fig. 1. Coding genes located on the first half of the plus strand (blue solid line) and on the second half of the complementary strand (purple solid line) were assigned to the leading strand, as their transcription proceeds in the same direction as the replication fork; the remaining genes were assigned to the lagging strand. DNA sequences of protein-coding genes were extracted from the genome sequences based on the genomic coordinates given in the NCBI annotation files. If a gene was located on the minus (complementary) strand (the purple line), its sequence was reverse-complemented.

### Assignment of 4s and NS sites

Coding sequences on each strand were first split into codons. START and STOP codons were excluded. The three nucleotides in each codon were then classified into 4s, NS and other sites according to the codon table (Supplementary Data 8).

### Observed skews

AT and GC skews were calculated for each of the five site categories (interoperonic regions, NS and 4s sites on leading strands; NS and 4s sites on lagging strands). These are referred to as ‘observed skews', and are denoted as *γ*_AT_ (for AT skew) and *γ*_GC_ (for GC skew):


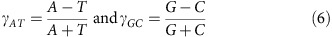


Where A, T, G, And C denote the numbers of the corresponding nucleotides in the class of sites considered (see Supplementary Data 3 for the data).

### Expression groups through tAI

tAI^23^,^24^ was used as a proxy for gene expression level. For each protein-coding gene in a given genome, tAI is defined as the average of tRNA availability values over all its codons. The availability of tRNAs for a codon considers not only the copy number of perfectly matched anticodons in the corresponding genome, but also that of imperfectly matched anticodons; the contribution of the imperfectly matched anticodons will be weighted accordingly. For more details on the definition of tAI see refs 23, 24. For each of the selected 1,550 genomes, we obtained a list of tRNA genes using the tRNAscan-SE^47^ programme on the genome sequences. The tRNA genes were sorted into 61 groups according to their anticodons. We then used the R scripts for tAI calculation written by dos Reis *et al*.^23^,^24^ (obtained from http://people.cryst.bbk.ac.uk/~fdosr01/tAI/, without modifications) to calculate tAI scores for all protein-coding genes in this genome. Higher tAI scores indicate higher expression levels.

Within each genome, coding genes on each strand (leading/lagging) were ranked according to their tAI scores from low to high and then divided into five equal-sized bins (quantiles), denoted Q1–Q5; Q1 contains the genes with the lowest, and Q5 contains the genes with the highest tAI scores. We then calculated skews at 4s and NS sites separately for each bin/quantile (see Supplementary Data 9 for the results).

### Levels of selection on nucleotide skew

Our model is rooted in the central dogma of genetics, that is, the information flow for protein-coding genes from genome to RNA to protein. One notable consequence of the central dogma is the single-stranded amplification of coding regions in transcription, with each resulting mRNA template amplified again single-strandedly by translation. Any selection responsible for skews in coding regions will be amplified accordingly.

If energy consumption is a fitness-relevant trait (which is likely the case for most prokaryotes), then cheaper nucleotides should generally be favoured by selection. For protein-coding nucleotides, the strength of this selection should be proportional to the number of mRNA copies that are produced during one cell cycle. For the same reason, cheaper amino acids should be favoured by translation, again in proportion to the number of protein copies produced.

For example, if in the coding region a G mutates to C, then this mutation will be favoured by selection on energy consumption. If this happens at a 4s site, the encoded amino acid will not change. However, if the same mutation happens at a NS site, it may be removed by translation-associated selection even if protein function is not affected, as the encoded amino acid may change to an energetically more costly alternative.

All three fundamental processes of the central dogma—replication, transcription and translation—contribute to biased substitutions. Non-transcribed (interoperonic) regions are only subject to replication; skews at 4s sites in coding regions are affected by both replication and transcription; and skews at NS sites are affected by all three processes. In the next section, we will model this interaction in the presence of genetic drift in finite prokaryotic populations.

### A mutation-selection-drift model for nucleotide skews

We assume that GC sites and AT sites are at an equilibrium, and only consider the mutations that convert A↔T and G↔C. The model is written up for GC skews; for the analogous model for AT skews, just replace GC with AT.

Let *u* be the mutation rate from C to G on the leading strand (caused by replication), and *v* the corresponding mutation rate from G to C, each measured per individual per generation. At transcribed sites, we need to combine mutational biases with selection on nucleotide and amino acid usage.

Consider a single genomic site that can be either C or G. Let *s* be the fitness difference between alleles G and C at that site; if only energetic cost affects fitness, then *s* corresponds to the total cost savings associated with a G allele relative to a C allele. Note that *s* incorporates selection on nucleotide usage (at all transcribed sites), as well as selection on amino acid usage (at *2s* and 1*s* sites): *s*=*s*_RNA_+*s*_AA_.

In a finite population, random sampling introduces stochastic fluctuations in allele frequencies, and we thus consider the equilibrium distribution of allele frequencies *f(p)* for the C allele^40^,^48^:


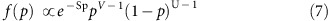


Here, *S*=2*N*_e_*s*, *V*=2*N*_e_*v*, *U*=2*N*_e_*u*, with the effective population size *N*_e_ (refs 49, 50). Mutation rates in prokaryotes are generally very small^17^, and are likely less than the inverse effective population size, 1/*N*_e_; thus, we have U+V<<1. In this situation, the allele frequency distribution is bimodal, such that the population will be either G or C at each site. The expected allele frequency *P* at a given site is then the probability of being C rather than G^18^:


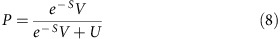


The derivation of equation (8) used the fixation probability of newly arisen mutants^50^ with *s*<1, 
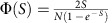
. The number of newly arisen C mutants that will eventually be fixed is 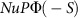, while the number of newly arisen G mutants that will ultimately be fixed is 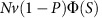; at equilibrium, these two rates are equal, leading to equation (8) (ref. 18).

Let *c* be the fraction of sites that contain C on the leading strand; *g*=1—*c* is the fraction of sites that contain G. *P* in equation (8) is the probability that a given site is C. Thus, if we consider a large number of sites that are either G or C, then *P*=*c*. The GC skew is defined as the fraction of G minus the fraction of C, that is, *γ*=g−c=1–2*c*; inserting equation (8), we obtain for the skew on the leading strand:


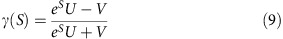


For the lagging strand, we need to reverse the roles of *u* and *v*, and we hence obtain


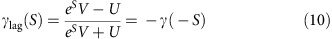


Note, that only ratios of mutation rates enter these equations. Thus, instead of the scaled mutation rates *U* and *V*, we can simply use the raw mutation rates *u* and *v*.

We define the relative skew in mutation rates as


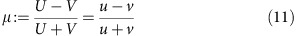


For non-transcribed sites, we assume that *S*=0, and equation (9) reduces to


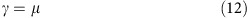


### Deriving the parameters from data

Let us define the ratio of the two mutation rates λ:=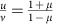. We can write


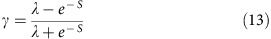


and, solving for *S*, we obtain


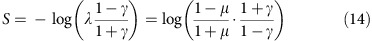


We obtain a corresponding equation for the lagging strand:


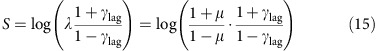


Combining (14) and (15) to eliminate *λ* allows us to relate the scaled selection coefficient *S* directly to the skews:


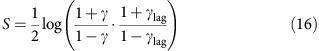


Conversely, eliminating *S* from equations (14) and (15), we obtain


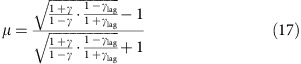


Transcription may influence the mutation rates *u* and *v*, so that the values in transcribed regions may differ from those of interoperonic regions; we call them *u*′ and *v*′ to distinguish them from the values at untranscribed sites. We assume that this influence is determined by basic cellular biology, and hence that 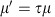 for some species-independent *τ* (which may depend on expression level). Replacing the mutational bias at transcribed sites with the scaled estimate from interoperonic regions, 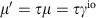, we can use the observed skews at 4s sites to estimate the scaled selection coefficient *S*=*S*_RNA_ (quantifying selection on nucleotide usage) separately for leading and lagging strands using equations (14) and (15).

In the same way, we can use equations (14) and (15) to estimate the scaled selection coefficient *S*=*S*_RNA_+*S*_AA_ at NS sites. NS sites differ from 4s sites predominantly in strong negative selection against some deleterious codon changes. Let us assume that we can classify sites into effectively neutral sites (those where an amino acid change is approximately selectively neutral) and fixed sites (where strong negative selection effectively prevents changes)^26^. If *f* is the fraction of effectively neutral sites, then the observed skew *γ* across all NS sites should be smaller by this fraction compared with the skew predicted by the model. Taking this into account, we replace the observed skew *γ*_NS_ by *γ*_NS_/*f*_*free*_ in the model equations for NS sites. Comparing the model-predicted *μ'* at NS sites with the value obtained from 4s sites, we can estimate the fraction of ‘free' sites *f*. Using this value, we can then estimate the scaled selection coefficient *S*=*S*_RNA_+*S*_AA_ at NS sites. Subtracting the scaled selection coefficient *S*_RNA_ for selection on nucleotide usage obtained from 4s sites, we obtain an estimate of selection on amino acid usage only: 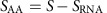.

### Random skews if there were no mutation and selection

To show the distributions of skews if there were no mutation and selection, we generated ∼1,200 random genomes with GC-contents ranging from 20 to 80%; for each genome, we generated about 1 million non-STOP codons using equal numbers of A and T, as well as G and C, and calculated the skews at 4s and NS sites. Any remaining skews are due to the nucleotide composition of the stop codons. The median values for AT skews are 0 at 4s sites and 0.018 at NS sites; for GC skews they are 0 and 0.081, respectively (see Fig. 3).

### The energy trade-off encoded in the codon table

To explore the relationship of the costs between nucleotides used in codons and the encoded amino acids, we performed a simulation study. We generated random coding sequences with given GC content (ranging from 10 to 90%) and equal AT and GC skews (ranging from −0.8 to 0.8). For each combination of the two parameters (GC content and skew), 1 million valid codons (excluding STOP codons) were generated, and the average cost of the encoded amino acids was calculated according to the bacterial codon table. The resulting values were used to generate Fig. 4, and can be found in Supplementary Data 10. Note that AT and GC skews contribute to the trade-offs independently, that is, the trade-offs still exist when the simulation was run using only AT skews (with GC skews set to 0) or only GC skews (with AT skews set to 0); see Supplementary Fig. 9 for the plots and Supplementary Data 11 and 12 for the data (please refer https://github.com/evolgeniusteam/nucleotideSkews/wiki for the source codes used for the simulations). The AA costs used here are from *E. coli*^5^, and might be slightly different in other species. Note that costs were calculated under the assumption that amino acids are synthesized from scratch; this is not always the case *in vivo*.

### Estimate of energy investments in *E. coli* metabolism

We obtained the genome-scale metabolic network of *E. coli* from Orth *et al*.^51^. This metabolic network model represents the most comprehensive and detailed knowledge about any genome-scale metabolic system. Using the *sybil* constraint-based analysis library^52^ for R, we performed flux-balance calculations in a glucose-limited aerobic minimal medium with the standard wild-type biomass reaction^51^. In this environment, glucose provides the only energy source. Similar results were obtained in the corresponding aerobic growth medium. We set the ‘maintenance ATP requirement' to 0.

### Estimation of maximal ATP production per molecule of glucose

To estimate how many molecules of ATP can be produced per molecule of glucose, we set a lower bound for the ATP→ADP reaction to 1 and minimized glucose uptake. We found that in this model, one molecule of glucose allows the production of 23.5 units of energy in the form of ATP; identical results were found for GTP.

### RNA nucleotide production costs

We next set the production of each RNA nucleotide (metabolites *ade, csn, gua, ura* in the iJO1366 model) in turn to ⩾1 and again minimized glucose uptake; the resulting solution gives the number of glucose molecules that need to be taken up to produce one RNA nucleotide molecule (Supplementary Data 1). To convert this estimate into an estimate of energy expenditure, we multiplied the result by the number of ATP energy units that can be produced per glucose molecule, 23.5.

### Amino acid production costs

We used the same strategy to estimate the cost of *de novo* production of each of the 20 canonical amino acids (data not shown).

### Nitrogen consumption of nucleotides

We calculated the number of nitrogen molecules required for the *de novo* synthesis of each of nucleotides using the genome-wide metabolic model of *E. coli*. The syntheses of one A, C, G and U require 5, 3, 5 and 4 molecules of nitrogen, respectively.

### Proportion of total cellular investment channelled into nucleotide and amino acid production

To estimate the amount of glucose needed to produce one unit of the full *E. coli* biomass, we set the lower bound of the biomass reaction to 1 and again minimized glucose consumption. We then repeated this procedure, but this time restricting the biomass reaction to its RNA nucleotide components and its amino acid components, respectively. We estimated the fraction of total cellular investment channelled into RNA nucleotides and amino acids, respectively, as the ratio between the glucose consumption for the respective biomass components and the glucose consumption for the full biomass.

The R codes and the genome-wide metabolic model of *E. coli* are available for download at: https://github.com/evolgeniusteam/nucleotideSkews/wiki.

### Identification of preferred codons

To check if the CUB in the 1,550 prokaryotic genomes could be affected by the preferred usage of cheaper nucleotides, we identified CUBs for each genome using a method previously described by Hershberg and Petrov^53^. Briefly, for each protein-coding gene in each genome a Nc value was calculated as the measure of the overall codon bias of the gene using the ENCprime program^54^; Nc ranges from 20 (the most biased) to 61 (the least biased). For each gene the frequency of each valid codon was also calculated. Then for each codon a Spearman's correlation was calculated between its frequencies across all coding genes in a specific genome and the Nc values of the corresponding coding genes. A preferred codon would be then selected for each codon family (a codon family contains all codons that code for the same amino acid) if it has the strongest and significant negative correlation value among all codons within this codon family, and a Spearman's *P* value<0.05 per *n*, where *n* is the number of codons in this codon family. A Java implementation of this method is available at https://github.com/evolgeniusteam/nucleotideSkews/wiki; see Supplementary Data 13 for the preferred codons for selected genomes used in this study. In this study we limited our analyses to 4s codons.

### Testing our model on 344 non-redundant bacterial genomes

To exclude potential biases resulting from an uneven phylogenetic distribution of the 1,550 genome sequences obtained from the NCBI database, we repeated our analyses on a subset of phylogenetically non-redundant bacterial genomes. This genome set was manually curated by Wu *et al*.^19^. Out of the 364 genomes selected by Wu *et al*.^19^, 344 overlapped with our study (Supplementary Data 2). Limiting our analyses to the 344 genomes, we obtained essentially identical results (Supplementary Figs 3–5).

### Accounting for phylogenetic relatedness

When calculating statistical significance across genomes, we need to account for the effects of phylogenetic relatedness: two genomic properties may be correlated just because they co-occur in phylogenetic clades because of their shared ancestry^21^. To control for the confounding effect of phylogenetic relatedness, we first needed to establish a phylogenetic tree (reflecting the signal of vertical inheritance) for the 1,550 genomes. For this, we followed the procedure described in ref. 55 with minor modifications. Briefly, we identified 40 universally conserved marker genes described in ref. 55 from each genome using fetchMG (http://vm-lux.embl.de/~mende/fetchMG/contact.html) and saved their protein sequences into 40 files, one for each marker gene. We aligned the protein sequences in each file using MUSCLE^56^ with parameter ‘-maxiters 100', eliminated divergent and ambiguously aligned regions from the resulting multi-sequence alignments (MSAs) using Gblocks^57^ with parameters ‘-t=p -b3=8 -b4=2 -b5=h' and concatenated all 40 MSAs. We then used RAxML^58^ to build a maximum likelihood tree with default parameters. We visually validated the tree using Evolview^48^, confirming that species from recognized clades largely clustered together. The identified marker genes for the 1,550 genomes, the concatenated MSA, and the resulting phylogenetic tree are available for download at https://github.com/evolgeniusteam/nucleotideSkews/wiki; the phylogenetic tree with colour-strips representing major taxonomic clades is shown in Supplementary Fig. 12. The maximum likelihood tree was then re-rooted between archaea and bacteria, making both domains monophyletic. The rooted tree was converted to an ultrametric tree using the *chronos*() function in the ape^59^ package for the *R* environment (https://www.r-project.org/) for scientific computing.

On the basis of this ultrametric tree, we used the caper *R* package (https://cran.r-project.org/web/packages/caper/) to convert all parameters to independent contrasts^21^ and performed the statistical tests on these.

### The effects of lifestyles on nucleotide skews

Intracellular bacteria rely mostly (if not completely) on their hosts for resources; we thus expect them to be under weaker selection for efficient resource usage. We selected 128 intracellular species (Supplementary Data 7; 116 of which overlapped with the 1,550 genomes used in our study) and 734 free-living species (those with existing habitat assignment that was other than ‘host-associated' according to the NCBI BioProject^43^; see Supplementary Data 3) for comparison. To ensure that estimates of scaled selection coefficients on transcribed sequences (*S*) were independent between genomes, we did not use a global amplification factor *τ* for the mutational bias; instead, we re-calculated the *z* values using estimates of mutational bias *μ*' directly obtained from the transcribed sequences of each genome. We applied general linear models (function *glm*() in *R*) to the phylogenetically independent contrasts^21^, relating the scaled selection coefficients *S*_RNA_ and *S*_AA_ for AT and GC skews to lifestyle (a binary factor, *INTRA*: intracellular or free-living), including genomic GC content (GC) and genome size (L) as potential confounding factors, and accounting for interactions between the predictors: *S* ∼ *INTRA* × *GC* × *L*. We removed non-significant terms until all terms (including interactions) were significant; each such model reduction decreased the AIC (Akaike Information Criterion) value. For *S*_RNA_(GC), all terms were statistically significant (*P*<0.05); for *S*_AA_ (GC) we removed the interaction between *INTRA* and *L*; and for *S*_RNA_(AT) and *S*_AA_ (GC) we removed the direct GC term, but kept the interactions involving GC.

The results show that all scaled selection coefficients are significantly weaker in prokaryotes with an intracellular lifestyle, consistent with our expectation. The R codes are available for download at https://github.com/evolgeniusteam/nucleotideSkews/wiki.

### The effects of flanking nucleotides on nucleotide skews

The frequencies of specific nucleotide triplets around 4s sites (that is, the 4s site plus its two neighbouring bases) are strongly correlated within genomes between leading and lagging strands (Pearson's *r>0.9*, *P*<10^−15^ in 93% genomes). Flanking nucleotides are known to influence the rates of specific mutations^33^. Thus, mutational biases associated with triplets predominantly found on transcribed protein-coding strands could in principle offer an alternative explanation for the skew contributions that we attributed to transcription-related selection. This alternative ‘genomic context' model posits that it is not transcription *per se*, but the nucleotide context found around 4s sites that is responsible for the characteristic skews found in transcribed regions.

To directly test the influence of genomic context, we assembled a data set that controls for the flanking nucleotides. We considered only nucleotides with ‘complementary symmetrical' neighbours, that is, those sites *x* for which the flanking nucleotides were identical to the flanking nucleotides of the paired base on the complementary strand: C*x*G, G*x*C or T*x*A (for example, A flanked by G and C—GAC—has the same genomic context as its paired base T—GTC). We then repeated our calculations on this subset of sites with complementary symmetrical neighbours. The genomic context model posits that all systematic within-genome variation in skews is caused by mutational biases due to genomic context. Thus, transcribed sites with the same flanking nucleotides on the leading strand should show the same skew, regardless of whether transcription occurs from the leading or from the lagging strand. Accordingly, the genomic context model predicts that *S*_RNA_ estimated for 4s sites with complementary symmetrical neighbours should vanish.

The data is available in Supplementary Data 14, and the results are shown in Supplementary Fig. 11.

## Additional information

**How to cite this article:** Chen, W.-H. *et al*. Energy efficiency trade-offs drive nucleotide usage in transcribed regions. *Nat. Commun.* 7:11334 doi: 10.1038/ncomms11334 (2016).

## Supplementary Material

Supplementary Information Supplementary Figures 1-13

Supplementary Data 1 Energy usage for the de-novo production of RNA nucleotides. These values were calculated using the metabolic model of E. coli (see Methods).

Supplementary Data 2 non-redundant bacterial genomes manually selected by Wu *et al*. (PMID: 22230424). Note: 364 genomes were manually selected by Wu and colleagues, among which 344 overlapped with the 1550 genomes we used in this study.

Supplementary Data 3 Prokaryotic organisms used in this study and numbers of nucleotides (ATGC) in interoperonic regions and at fourfold- (4s), twofold- (2s), and nonsynonymous (ns) coding sites, stratified by leading and lagging strands. The column names use the following abbreviations: 1 = ns site, 2 = 2s site, 4 = 4s site; le = leading strand, la = lagging strand. Thus, "A1le" indicates the number of A's at non-synonymous sites on the leading strand, etc.

Supplementary Data 4 Cost of amino acid synthesis; the data were obtained from different sources (PubMed IDs: 11904428 and 22538926).

Supplementary Data 5 GC content and average cost of encoded amino acids of proteincoding genes on leading (Le) and lagging (La) strands, stratified by tRNA adaptation index (tAI). Coding genes on each strand are ordered according to increasing tAI scores, and then divided into five approximately equally sized groups. Higher tAI scores indicate higher expression levels.

Supplementary Data 6 Replication origins used in this study. Data obtained from the DoriC database (http://tubic.tju.edu.cn/doric/).

Supplementary Data 7 Manually collected intracellular bacteria and those that are less than 750kb in size (see Methods for details).

Supplementary Data 8 Codons and encoded amino acids together with the degeneracy per codon position. The column "114" indicates that the first and the second codon positions are nonsynonymous (ns) sites, while the third codon position is a fourfold-synonymous (4s) site, etc.

Supplementary Data 9 Observed skews of coding regions on leading (le) and lagging (la) strands, stratified by tRNA adaptation index (tAI). The column name "ATle1" means AT skews at ns sites of the leading strand, etc.: le = leading strand, la = lagging strand; 1 = ns sites, 2 = 2s sites, 4 = 4s sites. AT skew = (A-T)/(A+T), GC skew = (G-C)/(G+C) where ATGC are the numbers of the respective nucleotides.

Supplementary Data 10 The trade-off between the cost of nucleotides and encoded amino acids. See Methods for details. The source code used to generate this data can be obtained from https://github.com/evolgeniusteam/nucleotideSkews/wiki. The column name "AT1" means AT skews at ns sites. "overallSkews" refers to the overall AT and GC skews in the coding region (in valid, i.e., non-stop, codons). Column 'aaAvgCost' was calculated using amino acid cost data from PubMed ID: 11904428. The other four columns, 'aaAvgCost_Aer_Het', 'aaAvgCost_An_Het', 'aaAvgCost_Aer_Phot', 'aaAvgCost_An_Phot' were based on amino acid cost data from PMID: 22538926 under aerobic heterotroph, anaerobic heterotroph, aerobic phototroph, and anaerobic phototroph lifestyles, respectively.

Supplementary Data 11 The trade-off between the cost of nucleotides and encoded amino acids; the simulation was run with AT skews only (GC skews were set to 0). The source code used to generate this data can be obtained from https://github.com/evolgeniusteam/nucleotideSkews/wiki. The amino acid cost data were obtained from PubMed ID: 11904428.

Supplementary Data 12 The trade-off between the cost of nucleotides and encoded amino acids; the simulation was run with GC skews only (AT skews were set to 0). The source code used to generate this data can be obtained from https://github.com/evolgeniusteam/nucleotideSkews/wiki. The amino acid cost data were obtained from PubMed ID: 11904428.

Supplementary Data 13 Preferred codons in the 1550 genomes.

Supplementary Data 14 Raw skews for 4s and interoperonic sites, stratified by strands and flanking nucleotides.
