# Supplementary material for: Energy efficiency trade-offs drive nucleotide usage in transcribed regions
Source: Nat Commun. 2016 Apr 21;7:11334. doi: 10.1038/ncomms11334 (PMC4844684; doi:10.1038/ncomms11334)
Supplement: Supplementary Information — Supplementary Figures 1-13 [file ncomms11334-s1.pdf]

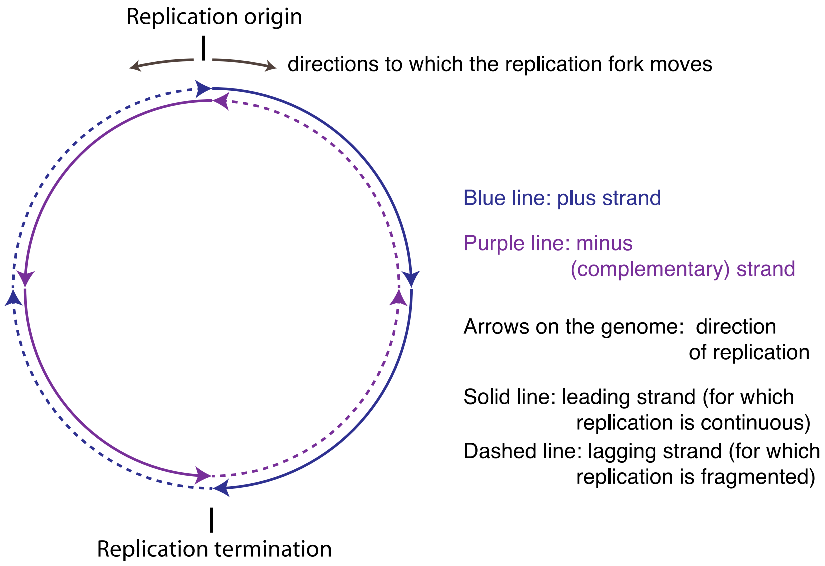

Supplementary Figure 1. The division of the genome into leading and lagging strands. Replication termination sites were calculated as the origin plus half the genome size.

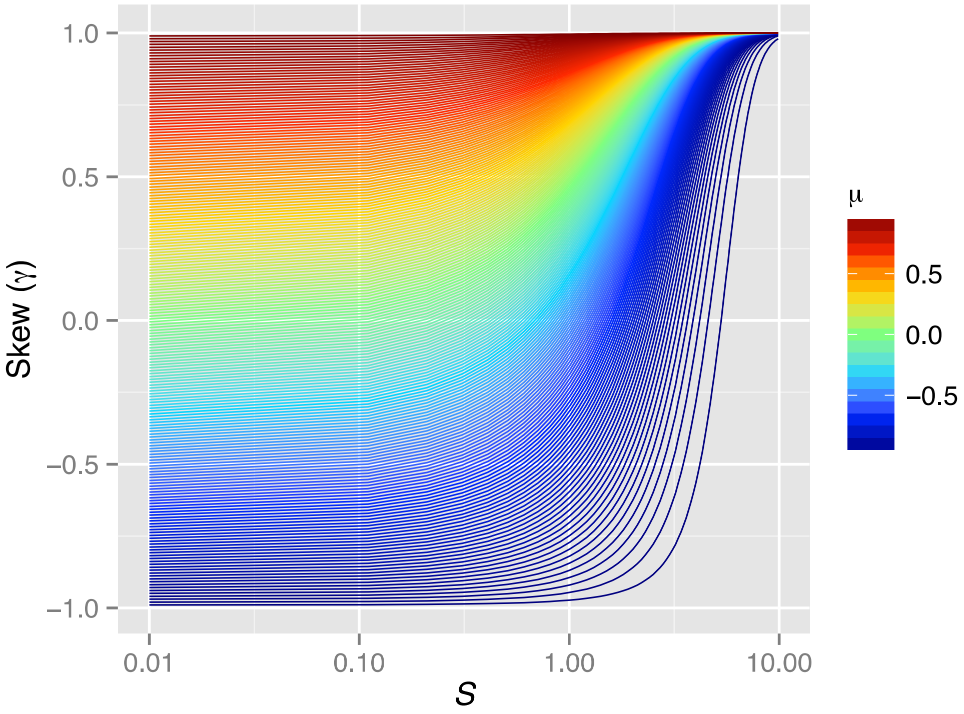

Supplementary Figure 2. A simulation plot illustrating the expected dependence of γ on S=$2N_e s$ and μ. The Y-axis shows the skews (γ values), while the X-axis shows the S values. Different colors correspond to different mutational (replication-associated) skews μ.

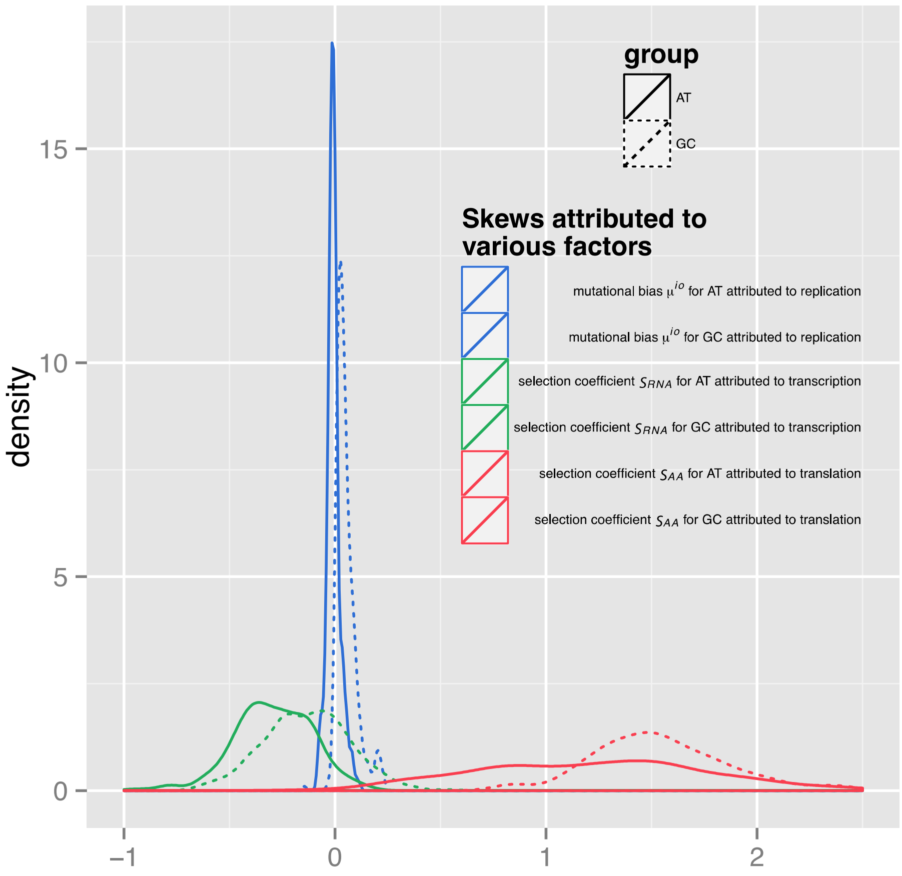

Supplementary Figure 3. Distribution of mutational bias (green) and scaled selection coefficients for *A* versus *T* (solid lines) and for *G* versus *C* (dotted lines) across 344 prokaryotic genomes. Similar to Figure 1, but showing results obtained from a subset of 344 phylogenetically evenly distributed prokaryotic species; see Methods for details and Supplementary Data 2 for the included strains. Blue: relative difference in mutation rates, μ^{io}, derived from inter-operonic sites; green: scaled selection coefficient related to transcription, S_{RNA}, derived from fourfold synonymous (*4s*) sites; red: selection related to translation, S_{AA}, derived from nonsynonymous (*ns*) sites. Replication-related selection favors the cheaper nucleotides *T/U* and *C* in most genomes ($S_{RNA}< 0$), while translation-related selection almost always favors the more expensive *A* and *G* ($S_{AA}> 0$). The scaled selection coefficients were averaged over the estimates from leading and lagging strand for each genome.

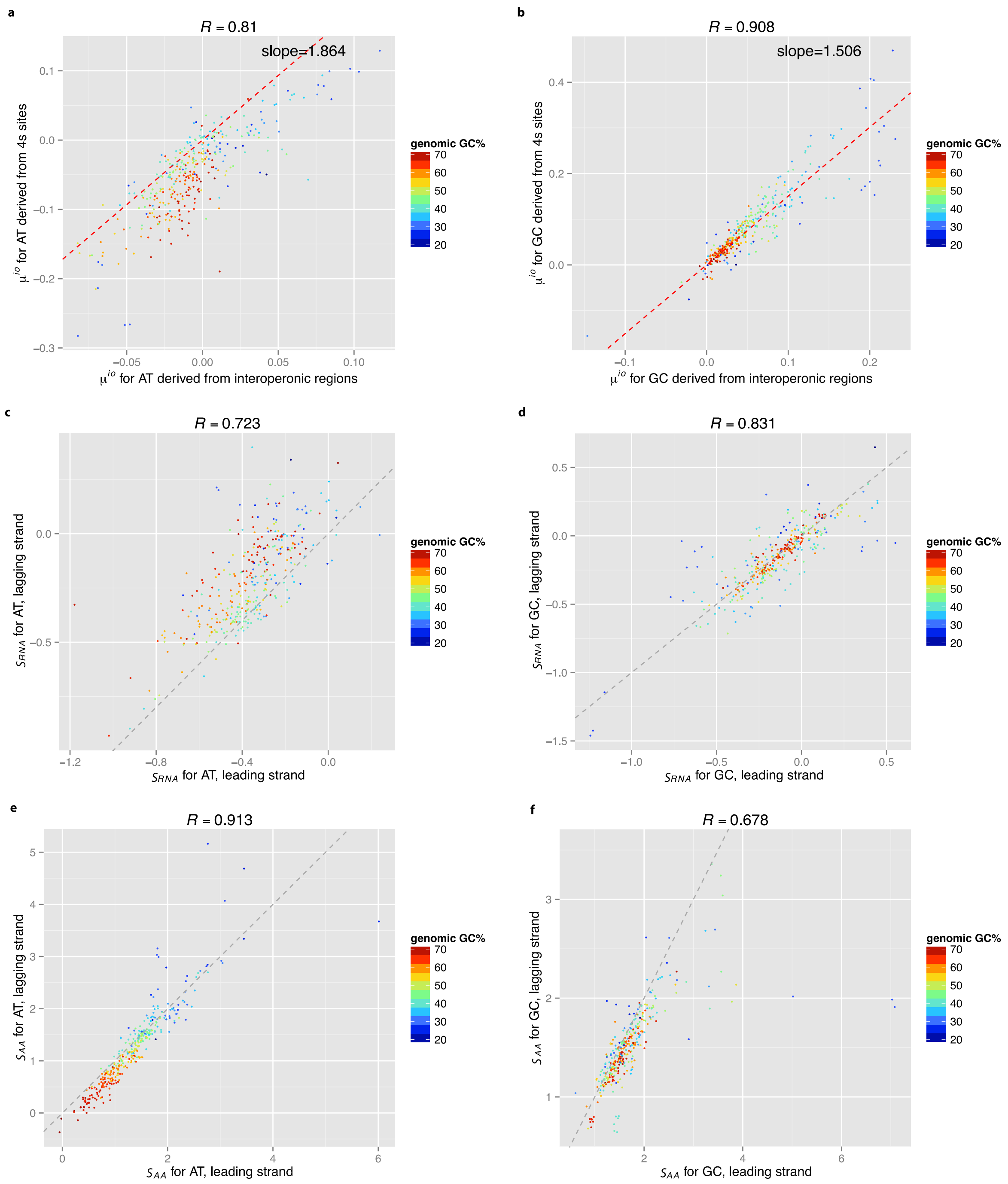

Supplementary Figure 4. Mutational biases attributed to replication **(a, b)** and scaled selection coefficients attributed to transcription **(c, d)** and translation **(e, f)** across 344 genomes, dissected using a mutation-selection equilibrium model. Similar to Figure 2, but restricted to a subset of 344 phylogenetically evenly distributed prokaryotic species; see Methods for details and Supplementary Data 2 for the included strains. Each point represents one genome, color-coded based on the genomic GC content. The red diagonal lines in A and B were obtained using linear regression through the origin; the grey diagonal lines in C-F indicate the expected identity of the two estimates.

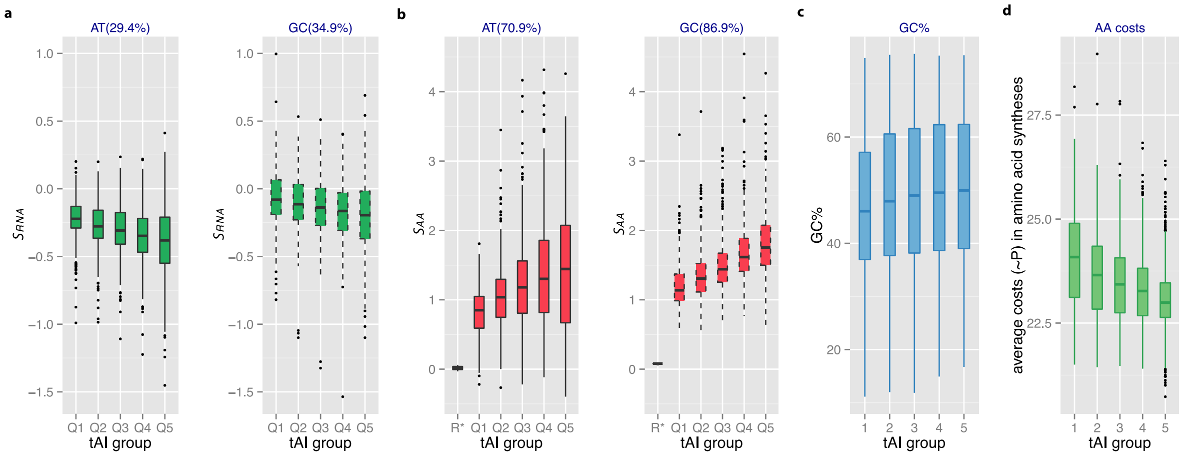

Supplementary Figure 5. Selection becomes stronger with increasing expression level (measured by tRNA adaptation index, tAI). Similar to Figure 3, but restricted to a subset of 344 phylogenetically evenly distributed prokaryotic species; see Methods for details and Supplementary Data 2 for the genomes. Scaled selection coefficients on the usage of expensive nucleotides attributed to transcription **(a)** become more negative with increasing expression level, while those attributed to amino acid usage **(b)** increase with increasing expression level. Highly expressed genes also tend to have higher GC content **(c)**. Accordingly, average amino acid energy costs **(d)** are much lower in more highly expressed genes. Q1-Q5 contain equal numbers of genes, grouped by expression levels from low to high. Percentages in parentheses give the fraction of genomes in which the skew of genes in the highest expression group (Q5) is higher than that of genes in the lowest expression group (Q1). R* shows the expected distribution for a random dataset (see Methods for details). This figure shows data averaged across leading and lagging strands. The data required to generate these plots can be extracted from Supplementary Datas 3 and 5 using the accession numbers in Supplementary Data 2.

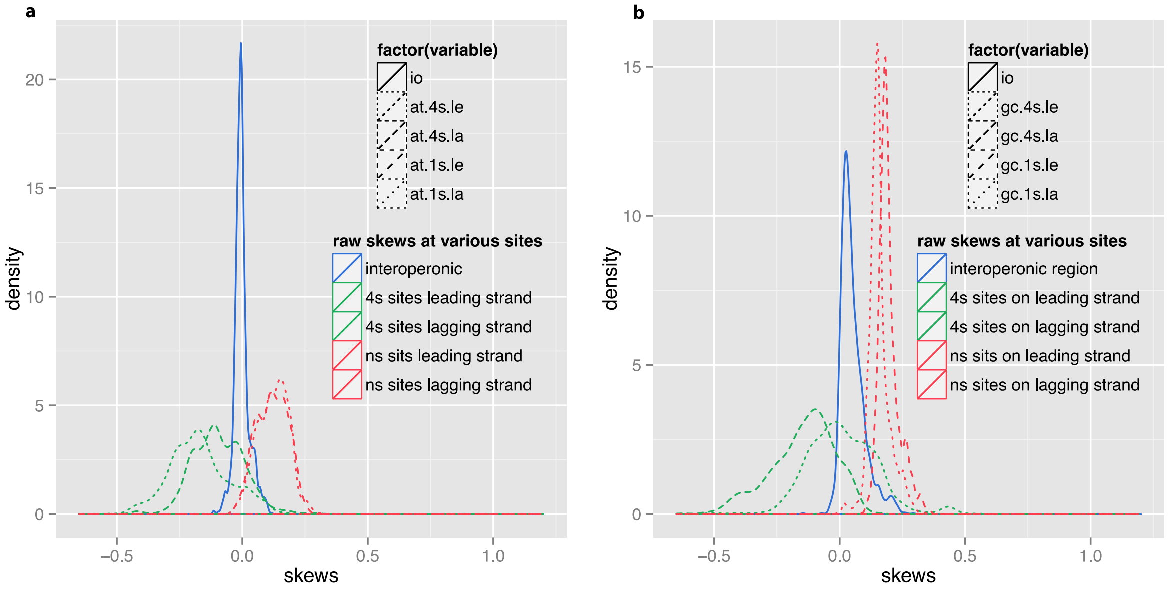

Supplementary Figure 6. Density plot of observed skews at five different types of sites. **(a)** AT skews; **(b)** GC skews.

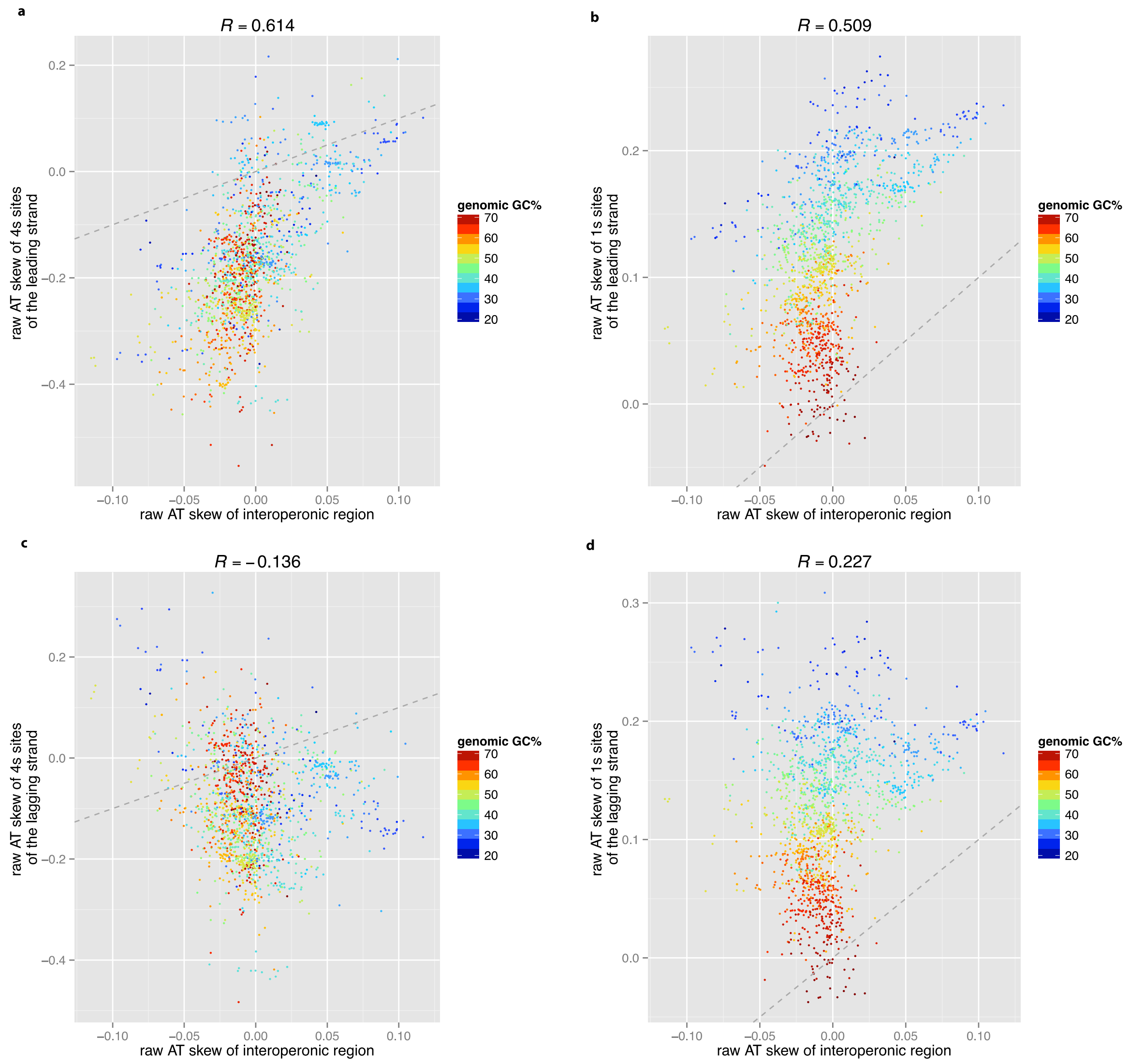

Supplementary Figure 7. Scatter plot of observed AT skews at different types of sites. The X-axis shows the raw skews in inter-operonic regions, while the Y-axes of the four panels show the raw skews of the leading strand at *4s* sites **(a)**, the lagging strand at *4s* sites **(b)**, the leading strand at *ns* sites **(c)** and the lagging strand at *ns* sites **(d)**, respectively.

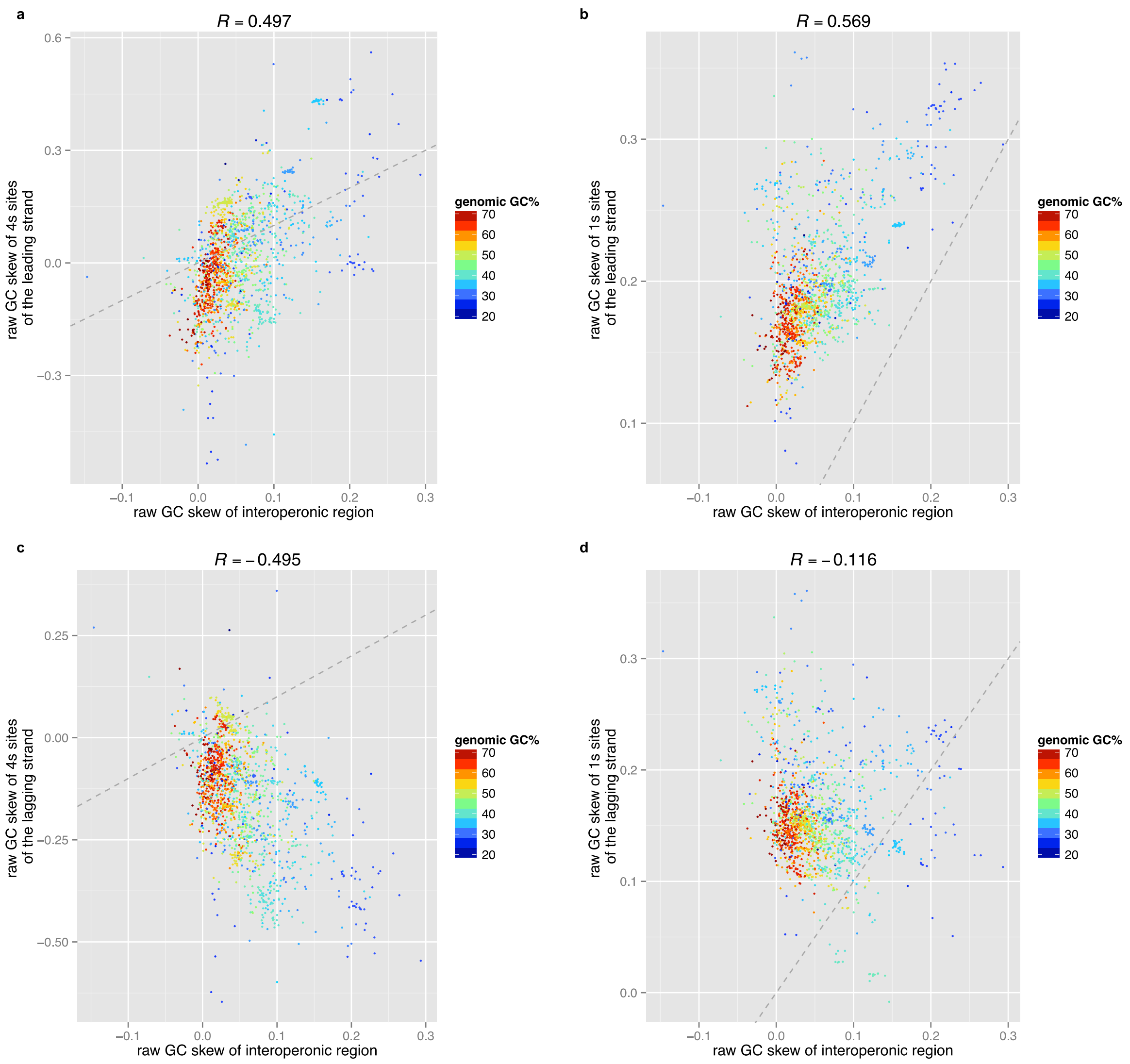

Supplementary Figure 8. Scatter plot of observed GC skews at different types of sites. The X-axis shows the raw skews at inter-operonic regions, while the Y-axes of the four panels show the raw skews of the leading strand at *4s* sites **(a)**, the lagging strand at *4s* sites **(b)**, the leading strand at *ns* sites **(c)** and the lagging strand at *ns* sites **(d)**, respectively.

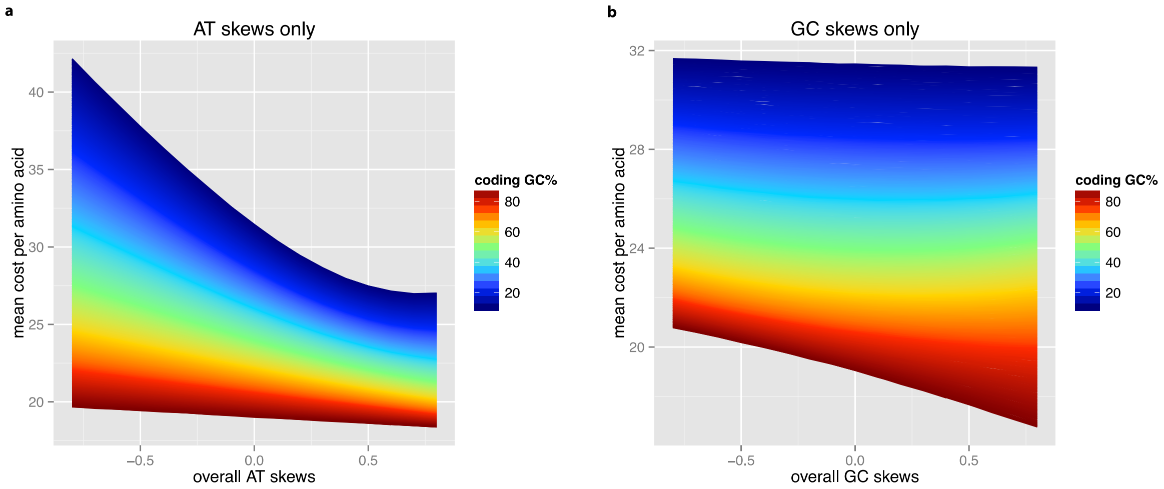

Supplementary Figure 9. AT and GC skews contribute independently to the tradeoffs. With AT skew only, the tradeoff between amino acid and nucleotide costs is still observable **(a)**; the same is true for GC skews only **(b)**.

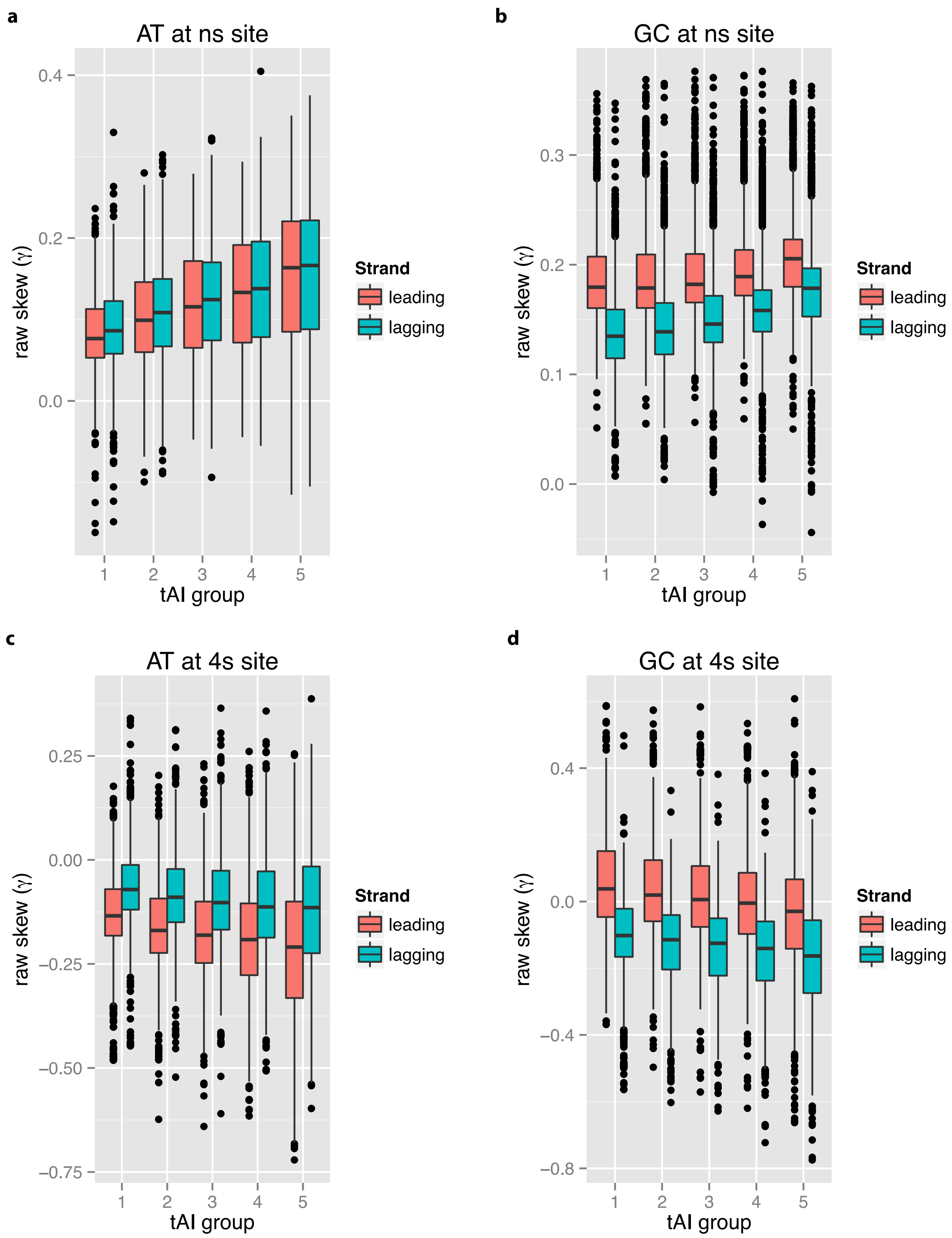

Supplementary Figure 10. Raw observed skews as a function of expression abundance. Groups 1-5 of the X-axis contain equal numbers of genes, grouped by tAI (as a proxy for expression levels) from low to high. Skews at non-synonymous (*ns*) sites for both AT **(a)** and GC **(b)** increase with increasing expression abundance, while skews at fourfold synonymous (*4s*) sites for both AT **(c)** and GC **(d)** decrease with increasing expression abundance.

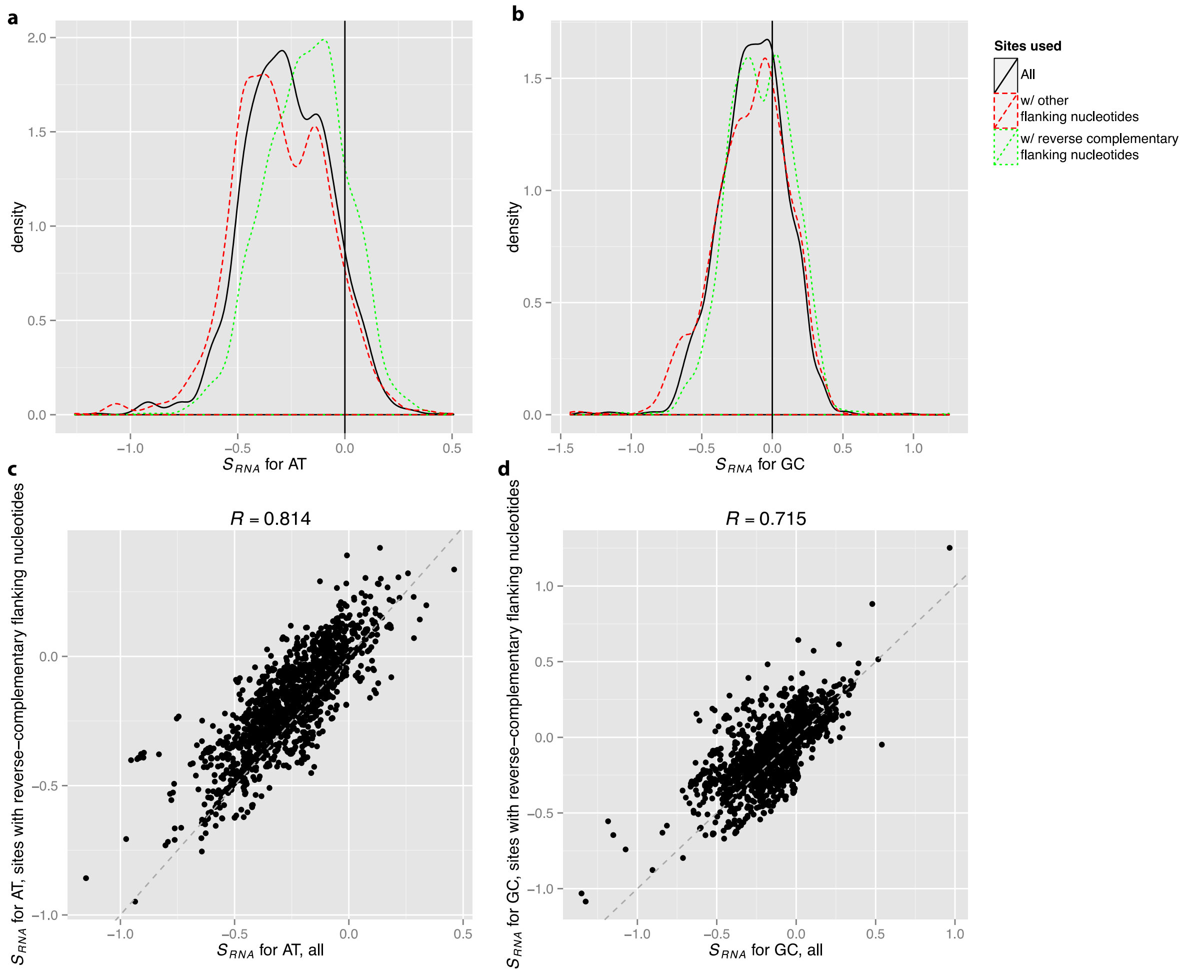

Supplementary Figure 11. Sites with complementary symmetric neighbors show transcription-associated selection coefficients (S_{RNA}) that are very similar to those estimated across all sites. Density plots of S_{RNA} values for sites with complementary symmetric neighbors (red), other neighbors (green) and all neighbors (black) are shown in **(a)** for AT sites and in **(b)** for GC sites. The correlations of S_{RNA} values between sites with complementary symmetric neighbors (Y-axis) and all sites (X-axis) are shown in **(c)** for AT sites and in **(d)** for GC sites.

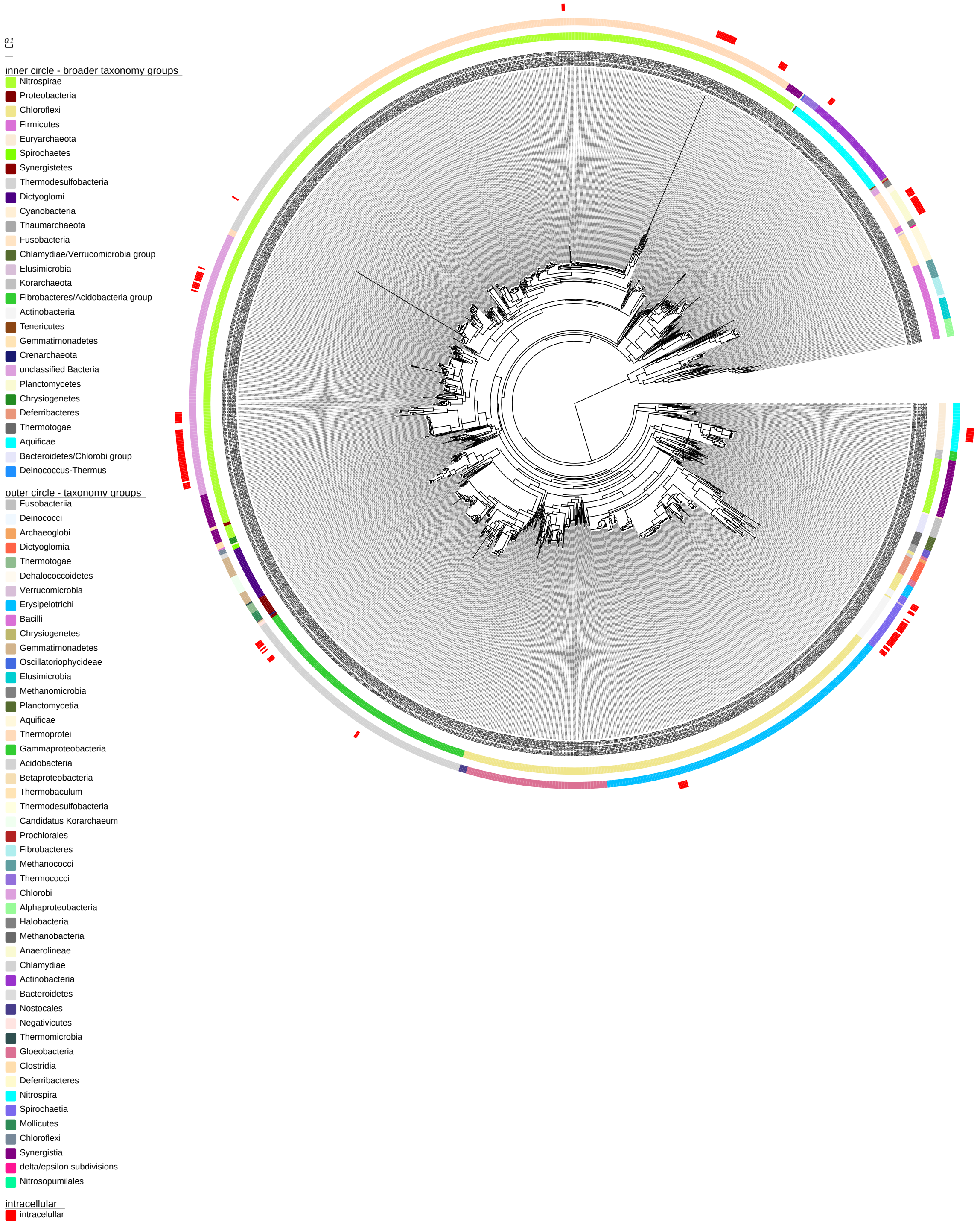

Supplementary Figure 12. A phylogenetic tree of the 1,550 prokaryotes used in this study, visualized using Evolview (http://www.evolgenius.info/evolview/). Organisms with their accession numbers highlighted in red are archaea. To show that species from major taxonomic clades can be positioned correctly on the phylogenetic tree, two color strips were plotted; in the inner strip, the 1,550 species were color-coded by the phylum (the ‘Group’ column in Supplementary Data 3) they are in, while in the middle strip, they were color-coded by the class (the “SubGroup” column in Supplementary Data 3) they are in. In addition, an outer strip was plotted to indicate the obligate intracellular organisms (pathogens and symbionts) used in this study. Note that the color-codes are independent in the three strips.

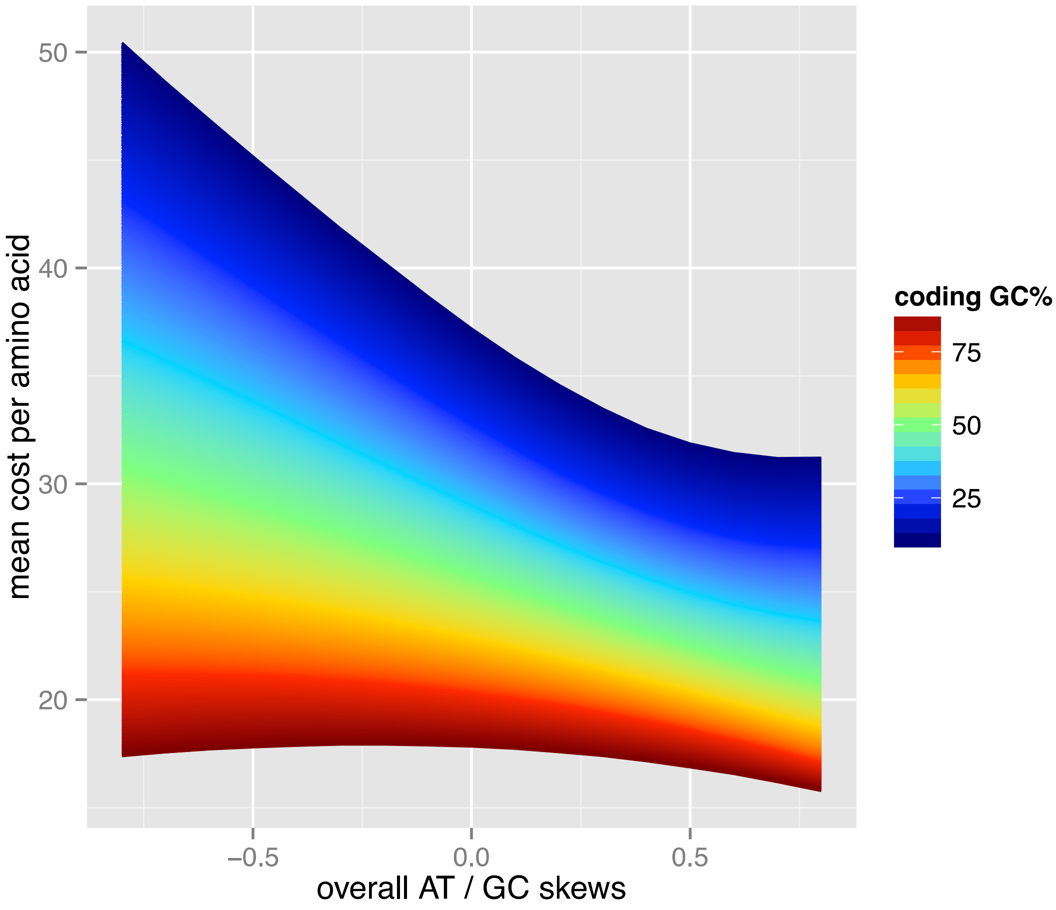

Supplementary Figure 13. The tradeoff between amino acid and nucleotide costs using an alternative source of amino acid costs. RNA nucleotide costs increase with increasing skews and GC%; conversely, amino acid costs decrease with increasing GC% and increasing skew. The same as Figure 4, but calculating amino acid costs based on an alternative source (see Supplementary Data 4, column 'aaAvgCost_Aer_Het').
